# Respiratory syncytial virus prolifically infects N2a neuronal cells, leading to TLR4 and nucleolin protein modulations and RSV F protein co-localization with TLR4 and nucleolin

**DOI:** 10.1186/s12929-018-0416-6

**Published:** 2018-02-10

**Authors:** Xiaoling Yuan, Tao Hu, Hanwen He, Huan Qiu, Xuan Wu, Jingxian Chen, Minmin Wang, Cheng Chen, Shenghai Huang

**Affiliations:** 1 Department of Microbiology, School of Basic Medicine, Anhui Medical University, Hefei, Anhui Province 230032 People’s Republic of China; 2 School of Life Sciences, Anhui Medical University, Hefei, Anhui Province 230032 People’s Republic of China; 3 Department of Laboratory Medicine, Anhui Health College, Chizhou, Anhui Province 247099 People’s Republic of China; 4 Department of Pathology and Cell Biology, Columbia University, New York, NY 10032 USA; 5 Department of Clinical Medicine, Anhui Medical University, Hefei, Anhui Province 230032 People’s Republic of China

**Keywords:** Respiratory syncytial virus (RSV), Toll-like receptor 4 (TLR4), Nucleolin (C23), Virus infection, N2a cells

## Abstract

**Background:**

Respiratory syncytial virus (RSV) infects the central nervous system, resulting in neurological symptoms. However, the precise underlying pathogenic mechanisms have not been elucidated. In the present study, the infectivity of RSV on N2a neuronal cells and the possible roles of Toll-like receptor 4 (TLR4) and nucleolin (C23) during RSV infection were investigated.

**Methods:**

We compared two experimental groups (infected and non-infected) and monitored the RSV viral titers in the culture supernatant by a viral plaque assay. We also inspected the morphology of the nucleus in infected N2a cells. We measured the level of RSV F protein and studied its co-localization with TLR4 and nucleolin using immunofluorescence assays and laser confocal microscopy. The potential interaction of RSV F protein with TLR4 and nucleolin was examined by coimmunoprecipitation. The expression changes of TLR4, nucleolin, TLR3 and TLR7 proteins in N2a cells and IL-6 and TNF-α in the culture supernatant were investigated by Western Blot analysis and ELISA assay. Changes in neuronal cell apoptosis status was examined by flow cytometry.

**Results:**

The results demonstrated prolific RSV infection of N2a cells, which triggered a decrease of NeuN protein expression, coinciding with an increase of nuclear lesions, F protein expression, RSV viral titers, and late apoptotic levels of N2a cells. RSV infection induced co-localization of RSV F protein with TLR4 and nucleolin, which could potentially lead to a direct interaction. Furthermore, it was found that TLR4 and nucleolin levels increased early after infection and decreased subsequently, whereas TLR3 and TLR7 expression increased throughout RSV infection.

**Conclusion:**

The RSV Long strain can prolifically infect N2a neuronal cells, modulating the expression of TLR4 and nucleolin, as well as TLR3, TLR7 and their downstream inflammatory factors, and inducing the co-localization of the RSV F protein with TLR4 and nucleolin.

## Background

Respiratory syncytial virus (RSV), an enveloped, single-stranded, negative-sense RNA pneumovirus, is a member of the *Orthopneumovirus* genus and *Pneumoviridae* family. Inflicting high morbidity and mortality rates in infants and young children, RSV is the most important pathogen responsible for lower respiratory tract infections in infants worldwide [1].

Recently, RSV was found to infect the nervous system and induce neurological symptoms, such as drowsiness, convulsions and epilepsy [2, 3], which might exist chronologically or independently. In clinical cases, acute encephalopathy was associated with RSV infection, and approximately 40% of RSV-positive patients presented with acute neurological symptoms before the age of 2 years [4]. Although the viral RSV genome has been detected in the cerebrospinal fluid (CSF) of children presenting with RSV infection-related convulsions or central nervous system (CNS) symptoms [5], the molecular mechanism underlying RSV neuropathogenesis remains unclear. Neuronal abnormalities might directly induce encephalopathic symptoms, such as epilepsy, seizures, convulsions or lethargy, and long-term neurological sequelae, including cognitive impairment and seizures, have been observed in patients. Additionally, movement disorders have also been observed in patients who survived encephalitis induced by Japanese encephalitis virus (JEV) infection [6].

N2a cells, a rapidly growing mouse neuroblastoma cell line, were derived from a spontaneous tumor in an albino strain A mouse. Because N2a cells differentiate into cells possessing many neuronal properties in vitro and express neuronal markers, such as neurofilaments, they are commonly used for studying neurotoxicity, Alzheimer's disease, and neurotropic viruses, such as HSV-1 and rabies virus [7–10]. N2a cells have also been employed as a model system to study the CNS pathology of JEV-induced viral encephalitis, as they are permissive to this virus [11]. Thus, N2a cells were selected to study the characteristics of RSV infection in the current study.

Nucleolin (C23), a ubiquitous 105-kDa nucleolar protein expressed in exponentially growing eukaryotic cells, is a cell surface receptor for several ligands, including midkine, matrix laminin-1, attachment factor J, and lipoproteins apo-B and apo-E [12–15]. This phosphoprotein is found mainly in the nucleus, where it regulates cell proliferation and growth, embryogenesis, cytokinesis, and nucleogenesis [16]. More recently, C23 was proposed to mediate the extracellular regulation of nuclear events [14] and shown to play roles in inflammatory responses induced by lipopolysaccharides (LPS) [17]. Furthermore, reports have also suggested that surface C23 may serve as an attachment target for some viruses, such as HIV [18, 19]. Based on its relative molecular mobility during sodium dodecyl sulfate-polyacrylamide gel electrophoresis (SDS-PAGE), C23 is often described as a 100–110-kDa protein, although its putative molecular weight is approximately 78 kDa [20]. Tayyari et al. reported that C23 acts as a cellular receptor to recognize the RSV F protein, and interfering with the C23-RSV F protein interaction inhibits RSV infection in cell cultures and in animal models [21]. These finding are consistent with the characteristics of other enveloped virus cellular receptors, such as those of herpes simplex virus [22] and hepatitis B virus [23].

Toll-like receptors (TLRs) play essential roles in innate immunity and are expressed in a wide range of cell types, including CNS cells. TLR engagement by specific sets of microbial ligands trigger the production of pro-inflammatory factors and enhance antigen-presenting cell functions. However, the functional roles of TLRs in the CNS are poorly understood [24]. Purified RSV F protein elicits an inflammatory response in hematopoietic cells, requiring the expression of TLR4 and its co-receptor CD14. While TLR4 expression is known to play an important role in controlling RSV replication [25], its biological role in immune responses to RSV remains inconclusive and controversial [26, 27].

F protein is a surface protein on RSV virions that mediates fusion of the virus membrane with that of the host cell, enabling RSV to enter cells and synthesize new F proteins in the infected cells [27]. Therefore, the RSV F protein plays a vital role in the pathogenesis of RSV infection. Kurt-Jones et al. reported that separate viral products, such as purified RSV F protein, might bind TLR4 and/or CD14, but no direct evidence supporting an interaction between intact RSV particles and the TLR4 receptor complex during the course of RSV pathogenesis have been reported [25].

Because neuronal cells express TLR4 and C23 [28, 29] and TLR4 and C23 are capable of recognizing the RSV F protein, we tentatively hypothesized that RSV might enter neuronal cells via interactions of the F protein with TLR4 and C23, resulting in the proliferative infection of N2a neuronal cells. Thus, in this study, the ability of RSV Long strain to infect N2a neuronal cells was investigated, and the relationships between TLR4 and C23 expression and the RSV F protein in N2a cells were explored. In addition, because TLR3/TLR7 might recognize double-stranded RNA and the single-stranded RNA genome during RSV replication, TLR3/TLR7 variations were also studied in RSV-infected N2a cells. Hopefully, our study will help elucidate new therapeutic targets for the prevention and control of human RSV-associated encephalopathy.

## Methods

### Cells and viruses

N2a neuronal cells (a gift from Prof. Yu-Xian Shen, Anhui Medical University, Hefei, China) were cultured in DMEM (Dulbecco’s Modified Eagle’s Medium, Life Technologies Corp, USA) supplemented with 10% fetal bovine serum (HyClone, USA) and 1% penicillin (100 U/ml)-streptomycin (100 μg/ml). The cells were incubated in a humidified atmosphere comprising 95% air and 5% CO_2_ at 37 °C.

RSV Long strain (a gift from Dr. Hai-Ming Wei, Institute of Immunology at the University of Science and Technology of China, Hefei, China) was propagated in HEp-2 cells, and viral stocks were purified and stored in liquid nitrogen. Viral titers were determined using a modified methylcellulose plaque assay as described by Kisch et al. [30] using confluent HEp-2 cells grown on 12-well plates (Corning Inc., USA). For the experimental infections, N2a cell monolayers were infected with RSV at a multiplicity of infection (MOI) of 2 in 24-well cell culture plates (Corning Inc.).

Similarly, RSV viral titers in N2a cells were determined by a modified plaque assay [30]. Briefly, N2a cells were first seeded in 12-well tissue culture plates at a density of 5 × 10^4^ cells per well in high-glucose DMEM supplemented with 10% heat-inactivated fetal calf serum (FCS) Supernatants from the N2a cell culture medium were collected at 1 h, 2 h, 4 h, 12 h, 24 h, 2 d and 4 d after RSV infection and then 10-fold serially diluted. Five hundred microliters of each diluted supernatant was added to the corresponding well. The plates were then incubated for 2 h at 37 °C with gentle shaking every 15 min. The medium was removed from the wells, and the primary overlay, consisting of 1% SeaKem agarose mixed with 2× DMEM (1:1) and 5% FCS, was added. The cultures were then incubated at 37 °C in 5% CO_2_ for the indicated times required for each assay. Next, 1 ml of crystal violet solution was added to each well, and the plates were incubated at room temperature (RT) for 1 h. Finally, the plates were washed with fresh tap water and air-dried, and the total plaques in each well were then counted.

The experimental groups of N2a cells were infected with RSV Long strain for varying durations, and normal cells were used as the uninfected control group.

### Antibodies and reagents

A goat polyclonal antibody against TLR4 (sc-16,240), a rabbit polyclonal IgG antibody (sc-28,999) against TLR3 (M-300), and a rabbit polyclonal IgG antibody (sc-30,004) against TLR7 (H-114) were purchased from Santa Cruz Biotechnology (TX, USA). A rabbit polyclonal antibody against C23 (ab22758), a mouse monoclonal antibody against F protein (RSV3216 (B016), ab24011), an anti-NeuN antibody (EPR12763, neuronal marker, ab177487), a donkey anti-goat IgG heavy and light chain (H&L) antibody (Alexa Fluor 555, ab150130) against TLR4, a goat anti-rabbit IgG H&L antibody (Alexa Fluor 488, ab150077) against C23, and a goat anti-mouse IgG H&L antibody (Alexa Fluor 647, ab150115) against RSV F were purchased from Abcam (Cambridge, UK). The Annexin V-FITC apoptosis kit (BB-4101) was obtained from BestBio (Shanghai, China), and enzyme-linked immunosorbent assay (ELISA) kits for IL-6 and TNF-α were obtained from Hermes Criterion Biotechnology (Vancouver, Canada).

### Indirect immunofluorescence assay and laser confocal microscopy

N2a cells were plated on poly-L-lysine-coated glass coverslips at a density of 5 × 10^5^ cells/well and cultured for 24 h in 6-well cell culture plates (Corning Inc.). After RSV inoculation, the cells were fixed with 4% formaldehyde for 30 min at RT. The cells were permeabilized with 0.3% Triton X-100 for 20 min, and non-specific binding sites were blocked by incubation with 10% bovine serum albumin (BSA) for 30 min. The cells were then incubated with specific primary antibodies, including an anti-NeuN antibody, an anti-RSV F protein antibody (1:300), an anti-TLR4 antibody (1:300), and an anti-C23 antibody (1:300), overnight at 4 °C. After washing with PBS (3 × 5 min), the corresponding secondary antibodies conjugated to Alexa Fluor 555 (1:300), Alexa Fluor 488 (1:300), or Alexa Fluor 647 (1:300) were applied at RT for 1 h. The cells were then stained with DAPI (1:1000, Beyotime, China) at RT for 10 min, followed by mounting and examination with a laser scanning confocal microscope (SP8-DM26000, Leica, Germany).

### Co-immunoprecipitation assay

Co-immunoprecipitation was performed using an immunoprecipitation kit (Thermo Scientific™ Pierce™ Co-Immunoprecipitation, Thermo Scientific Corp, USA) according to the manufacturer’s instructions. Briefly, an anti-RSV F protein antibody was immobilized to the AminoLink coupling resin in a spin column, which was placed in a collection tube and centrifuged. The flow-through was saved to verify antibody coupling. At 1 h post-infection (pi), 2 h pi, 4 h pi and 8 h pi, N2a cell lysates were prepared and centrifuged at 13,000×g for 10 min; the supernatants were then transferred to new tubes to determine the protein concentrations. The supernatants were pre-cleared with the corresponding control agarose resin before being applied to the immobilized antibody columns. The prey proteins were mixed with the resin and incubated by gentle mixing for 1–2 h or overnight at 4 °C, followed by gentle washing with lysis buffer three times. Finally, the proteins were eluted with elution buffer and boiled for 5 min before being subjected to Western blot analysis. Polyvinylidene fluoride (PVDF) membranes were blocked with a solution containing primary antibodies against TLR4 and C23. A control lacking the primary antibodies was incubated with anti-mouse IgG.

### Flow cytometry assay

N2a cell apoptosis was analyzed using an Annexin V-FITC/PI apoptosis detection kit (BestBio, Shanghai, China) according to the manufacturer’s instructions. Briefly, the cultures were washed twice with PBS and treated with EDTA-free trypsin for 5 min to dislodge the cells. Then, the cell suspensions were centrifuged at 300×g for 5 min at 2–8 °C. The pellets were resuspended in 400 μl of Annexin V binding buffer, and the cell concentrations were adjusted to 1 × 10^6^ cells/ml. After 10 μl of Annexin V-FITC staining solution was added, the cells were incubated at 2–8 °C for 15 min in the dark. Then, 10 μl of propidium iodide (PI) staining solution was added, and the cells were incubated in the dark for an additional 5 min at 2–8 °C. When all the procedures were completed, the cells were immediately analyzed on a BD flow cytometer (BD FACS Calibur, BD Inc., USA).

### Co-localization analysis

Co-localization is defined as an overlap in the physical distribution of two or more labeled structures within a 3-dimensional space. In the current study, co-localization of the RSV F protein with TLR4 and C23 was evaluated using an analytical tool from the LAS AF software package and analyzed using an intensity correlation coefficient-based method.

Regarding the correlation coefficients, intensity correlation coefficient-based co-localization analysis utilized a statistical method to evaluate the relationship between fluorescence intensities. A linear equation describing the relevance of the intensities in an image pair was calculated by linear regression. The slope of this linear approximation supplied the association rate of two fluorochromes, and the coefficient estimated the quality of the approximation. The experiments were run in triplicate.

### Western blot analysis

N2a cells were harvested for Western blot analysis at different time points after RSV infection, and uninfected normal cells served as the control group. Briefly, cells were washed with PBS and lysed in lysis buffer containing phenylmethylsulfonyl fluoride (PMSF). The mixtures were kept on ice for 30 min and then centrifuged at 13,400×g for 5 min. The supernatants were collected and subjected to TLR4, C23, TLR3 and TLR7 protein detection. Equal amounts of total protein from each sample were loaded, separated by 10% SDS-PAGE, and then transferred to 0.45-μm PVDF membranes (sc-296,042, Santa Cruz Biotechnology). The membranes were then blocked with PBS/0.1% Tween-20 containing 5% non-fat milk for 2 h at RT and probed with primary antibodies (1:500 dilution for the TLR4, C23 and TLR7 antibodies, 1:1000 dilution for the TLR3 antibody) at 4 °C overnight. The membranes were then incubated with horseradish peroxidase-conjugated anti-rabbit or anti-mouse secondary antibodies (1:10,000, Santa Cruz Biotechnology) for 2 h. Enhanced chemiluminescence (SuperSignal West Femto Substrate Kit, Thermo Scientific) was employed to develop the blots. Bands were detected using a Tanon 4500 automatic digital gel image analysis system (Tanon 4500, Shanghai, China). The relative intensity of each protein was normalized to that of β-actin and quantified using ImageJ software. Additionally, a β-actin monoclonal antibody (TA-09, ZSJG-BIO, Beijing, China) was used as the internal control.

### ELISA analysis

The concentrations of IL-6 and TNF-α in the culture supernatants were measured using commercial ELISA kits according to the manufacturer’s instructions.

### Statistical analysis

Data are presented as the mean ± SEM. One-way analysis of variance (ANOVA) followed by Fisher’s post hoc test were used to determine statistically significant differences between groups (SPSS 17.0, SPSS, Chicago, IL, USA). *p* < 0.05 was considered significant, and *p* < 0.01 was considered highly significant.

## Results

### Altered NeuN protein expression and nuclear lesions in RSV-infected N2a cells

**Fig. 1:**
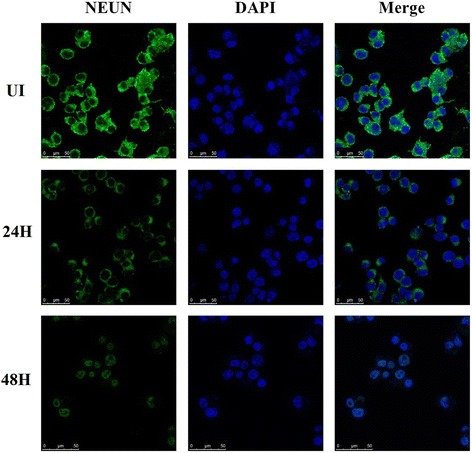
NeuN protein expression in N2a cells as determined by laser confocal microscopy. Normal and RSV-infected N2a cells were stained with an anti-NeuN antibody (green) and DAPI (blue, cell nuclei labeling) at 24 h pi and 48 h pi. UI indicates uninfected control cells

NeuN is a neuronal nuclear protein marker associated with cell death [31]. As shown in Fig. 1, uniform distribution and abundance of the NeuN protein was observed in intact, highly dense cells of the uninfected control group. However, in RSV-infected N2a cells, the number of NeuN-positive cells was decreased at 24 h pi and 48 h pi, with nucleus shriveling and cell death being observed.

**Fig. 2:**
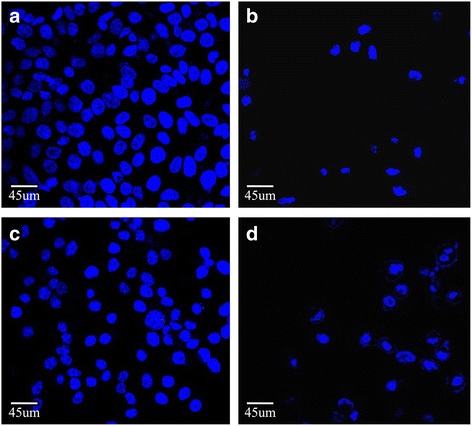
Nucleic changes in N2a and HEp-2 cells were observed by laser confocal microscopy using DAPI staining. **a** Nuclei of normal N2a cells; **b** nuclei of RSV-infected N2a cells at 4 d pi; **c** nuclei of normal HEp-2 cells; **d** nuclei of RSV-infected HEp-2 cells at 16 h pi

Additionally, changes in the nucleic morphologies of N2a cells between pre- and post-RSV infection were examined by laser confocal microscopy. Nucleic cell lesions were not apparent during the early infection period but became much more apparent during late infection. As shown in Fig. 2a, nuclei of RSV-infected N2a cells became irregular, fragmented or incomplete with decreasing nuclear densities at 4 d pi compared to that in uninfected N2a cells. In addition, many N2a cells unadhered from the plate 4 d after RSV infection (Fig. 2b). To confirm these changes, RSV-permissive HEp-2 cells were also subjected to testing using the same experimental procedure, and similar results were observed (Fig. 2c and d), demonstrating that RSV Long strain prolifically infects N2a cells and induces their death.

### Increased RSV F protein expression in RSV-infected N2a cells

**Fig. 3:**
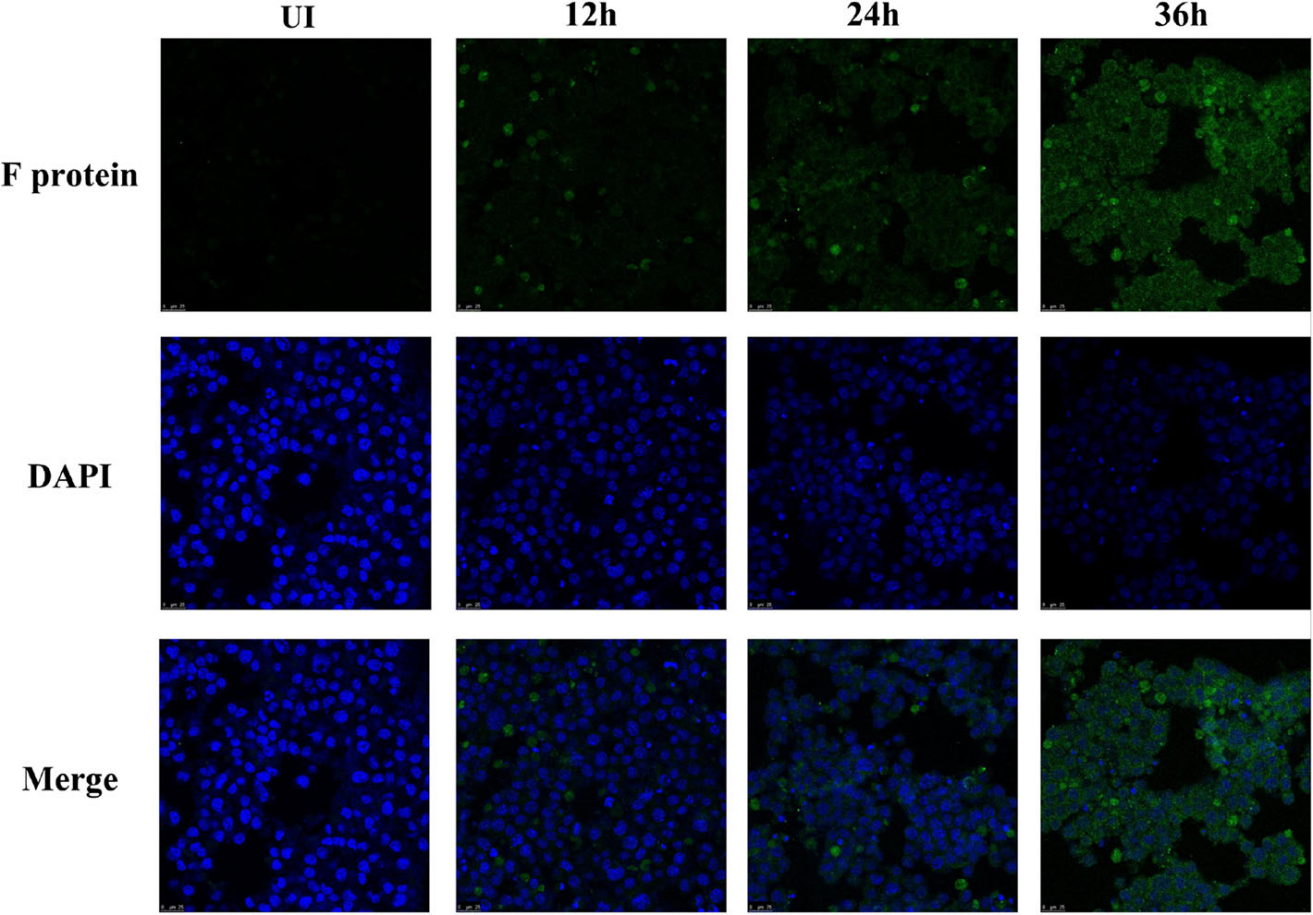
F protein expression levels in RSV-infected and uninfected N2a cells. RSV-infected and uninfected N2a cells were stained by an anti-F protein antibody at 12 h pi, 24 h pi, and 36 h pi and viewed under a fluorescence microscope; UI indicates the uninfected control. Nuclei were stained with DAPI and shown as blue fluorescence, and the RSV F protein was stained with a fluorescence-labeled antibody and shown as green fluorescence. The length of the infection was correlated with increasing RSV F protein expression (magnification, 400×)

As an RSV virion surface protein, F protein plays an important role in RSV fusion and entry and is also required for RSV propagation in vivo and in vitro. In the current study, RSV-infected N2a cells were assayed by immunofluorescence at different time points pi to detect F protein expression. F protein expression increased as the duration of infection extended (Fig. 3), suggesting that RSV can multiply in these neuronal cells.

### Increased viral titers in the culture supernatants of RSV-infected N2a cells

**Fig. 4:**
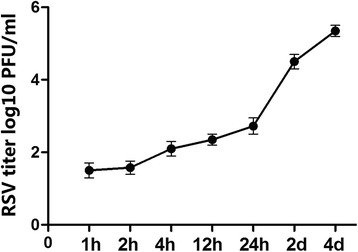
RSV viral titers in the culture supernatants of RSV-infected and uninfected N2a cells. Supernatants from each group of cells were collected at 1 h pi, 2 h pi, 4 h pi, 12 h pi, 24 h pi, 2 d pi, and 4 d pi to determine the viral titers (PFU) using the RSV plaque assay. Their numeric units are represented as log10 PFU/ml. Data are expressed as the mean ± SEM, and all tests were run in triplicate

In this experiment, N2a cells were inoculated with RSV at an MOI of 2 and cultured for 1 h, 2 h, 4 h, 12 h, 24 h, 2 d and 4 d. At the indicated time points, the viral titers in N2a cell culture supernatants were determined by a plaque assay [30]. Figure 4 shows the upward trend in the mean values of RSV plaque-forming units (PFUs) in the supernatants, demonstrating that RSV replicated in N2a cells in a time-dependent manner. Furthermore, replication was substantially more efficient between the 24 h and 4 d time points.

### Co-localization of the RSV F protein with TLR4 and C23

The membrane receptors TLR4 and C23 reportedly recognize the RSV F protein. To further understand its function and reveal early molecular events induced by viral infection, co-localization of the RSV F protein with both TLR4 and C23 were investigated using confocal microscopy.

**Fig. 5:**
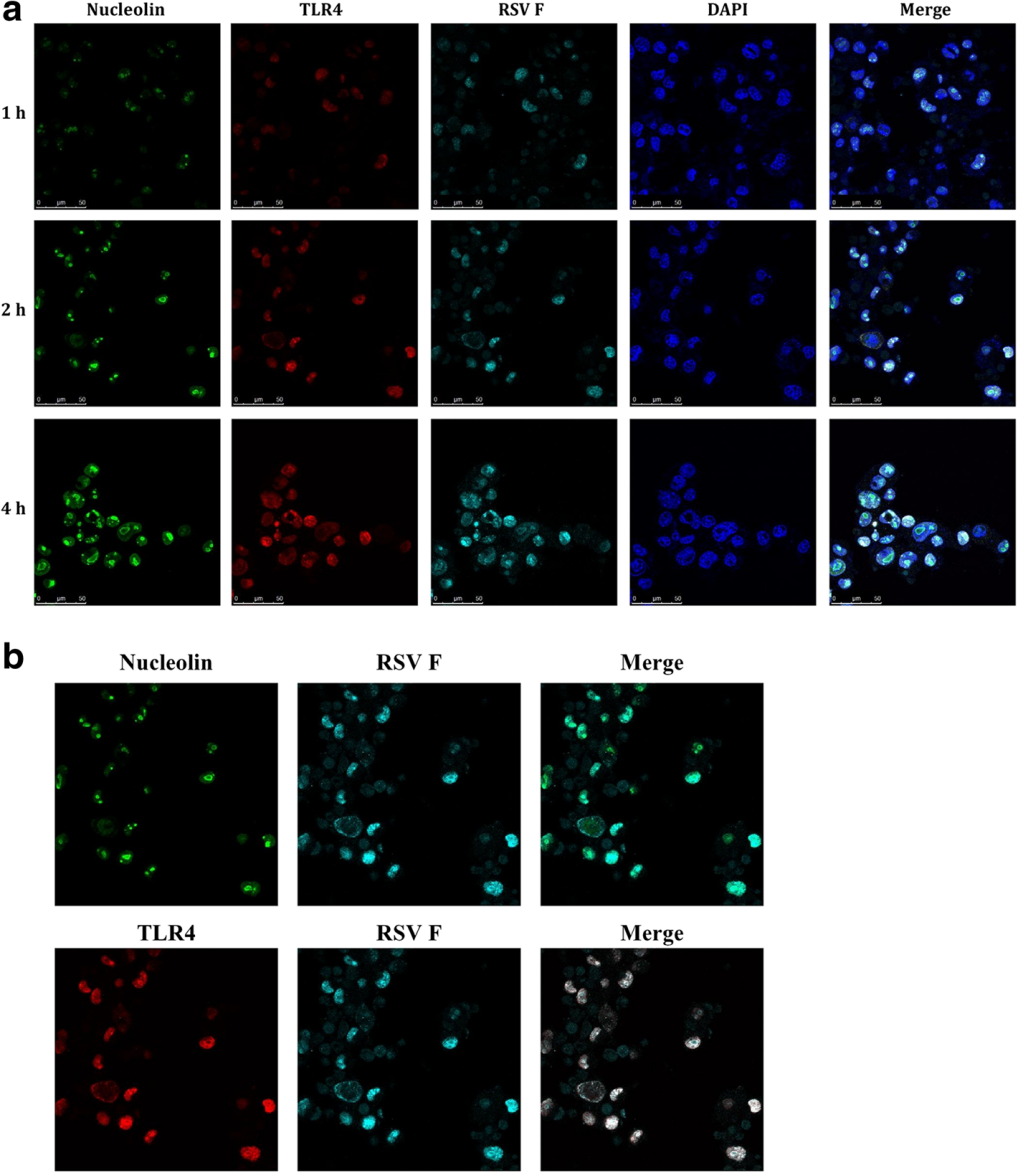
Co-localization of the RSV F protein with the cell surface receptors TLR4 and nucleolin and internalization of the RSV F protein into N2a cells were examined by laser confocal microscopy. N2a cells were fixed and stained with fluorescence-labeled antibodies against TLR4 (red), nucleolin (green) and the RSV F protein (cyan) at different time points after RSV infection. Simultaneously, N2a cell nuclei were counterstained with DAPI. **a** The RSV F protein co-localized with both TLR4 and nucleolin at 1 h pi, 2 h pi and 4 h pi; **b** The RSV F protein co-localized with TLR4 and nucleolin in N2a cells at 2 h pi; nucleolin is labeled with Alexa 488 (green fluorescence), TLR4 is labeled with Alexa 555 (red fluorescence), and F protein is labeled with Alexa 647 (cyan fluorescence). Co-localization was evaluated using an analytic tool from the LAS AF software package

After inoculation with RSV, N2a cells were stained with fluorescence-labeled antibodies against the RSV F protein (cyan), TLR4 (red) and C23 (green) at 1 h, 2 h and 4 h pi. F protein expression and RSV proliferation in N2a cells increased in a time-dependent manner (Fig. 5a). As shown in Fig. 5b, the RSV F protein both co-localized and exhibited a correlative intensity with TLR4 and C23. These results were consistent with the findings of Tayyari et al. that C23 is recognized by the RSV F protein [21] and Rallabhandi et al. that the TLR4 signaling pathway is activated by the RSV F protein [27].

### Potential interactions of the RSV F protein with TLR4 and C23 detected by co-immunoprecipitation

**Fig. 6:**
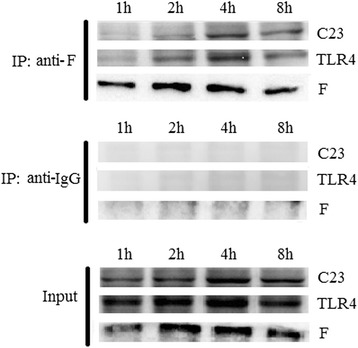
The potential interactive effects of TLR4 and nucleolin with the RSV F protein were assessed using a co-immunoprecipitation assay. The TLR4 and nucleolin proteins were pulled down with F protein, and their coalition in N2a cells was augmented after RSV infection but not throughout the entire infection period. Crude extract (input) and immunoenriched (IP) fractions were subjected to Western blot analysis. Tagged proteins were detected using an anti-RSV F protein antibody. Anti-mouse IgG served as the negative control

Immunoprecipitants containing prey proteins (TLR4 and C23), which potentially interact with the F protein, were pulled down using an anti-F protein antibody and then subjected to Western blot analysis. As shown in Fig. 6, TLR4 and C23 immunoprecipitation was significantly increased from 1 h pi to 4 h pi but decreased thereafter. A similar immunoprecipitation phenomenon was observed with C23. In the negative control group, lacking primary antibodies against TLR4 and C23, no bands were detected by the co-immunoprecipitation assay using anti-mouse IgG.

### Altered TLR4 and C23 protein expression levels in RSV-infected N2a cells

**Fig. 7:**
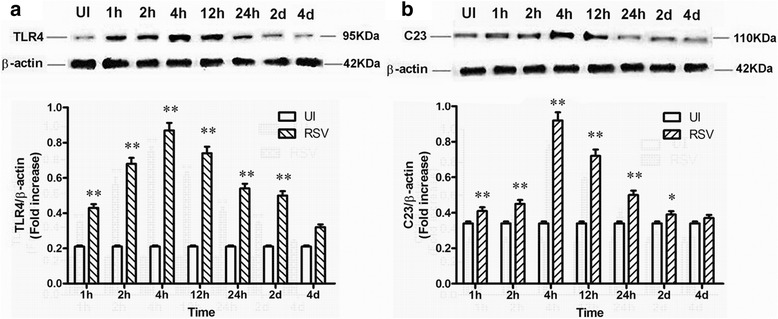
TLR4 and nucleolin protein expression in RSV-infected and uninfected N2a cells was measured by Western blot analysis. UI indicates the uninfected control; lanes 2–8 represent N2a cells at 1 h pi, 2 h pi, 4 h pi, 12 h pi, 24 h pi, 2 d pi, and 4 d pi, respectively. TLR4 (**a**) and nucleolin (**b**) protein expression levels were enhanced by infection durations less than 4 h pi and suppressed by infection durations greater than 4 h pi. The relative fold changes and statistical analyses of TLR4 (**a**) and nucleolin (**b**) expression levels were evaluated using GraphPad Prism 5 software (GraphPad Software Inc., La Jolla, CA, USA). Data are expressed as the mean ± SEM, and three independent experiments were run. ^*^P < 0.05 was considered statistically significant, and ^**^P < 0.01 was considered highly statistically significant compared with the uninfected control

TLR4 and C23 expression was examined by Western blot analysis at the indicated time points pi (Fig. 7). In the RSV-infected group, expression of the TLR4 protein was very low at 0 h pi, significantly increased from 1 h pi to 4 h pi, (*P* < 0.01), and decreased from 12 h pi to 4 d pi. Although a decrease in expression was observed, the expression level of TLR4 from 12 h pi to 2 d pi in the RSV-infected group remained higher than that in the uninfected control group (*P* < 0.01). However, the differences were not significant at the 4 d pi time point.

The variation tendencies of C23 protein expression were similar to those of TLR4. The difference in C23 expression between the RSV-infected group and the uninfected control group was statistically significant prior to 2 d pi (*P* < 0.01 and *P* < 0.05, respectively) but became statistically insignificant at the 4 d pi time point.

### Changes in the apoptosis of RSV-infected N2a neuronal cells

**Fig. 8:**
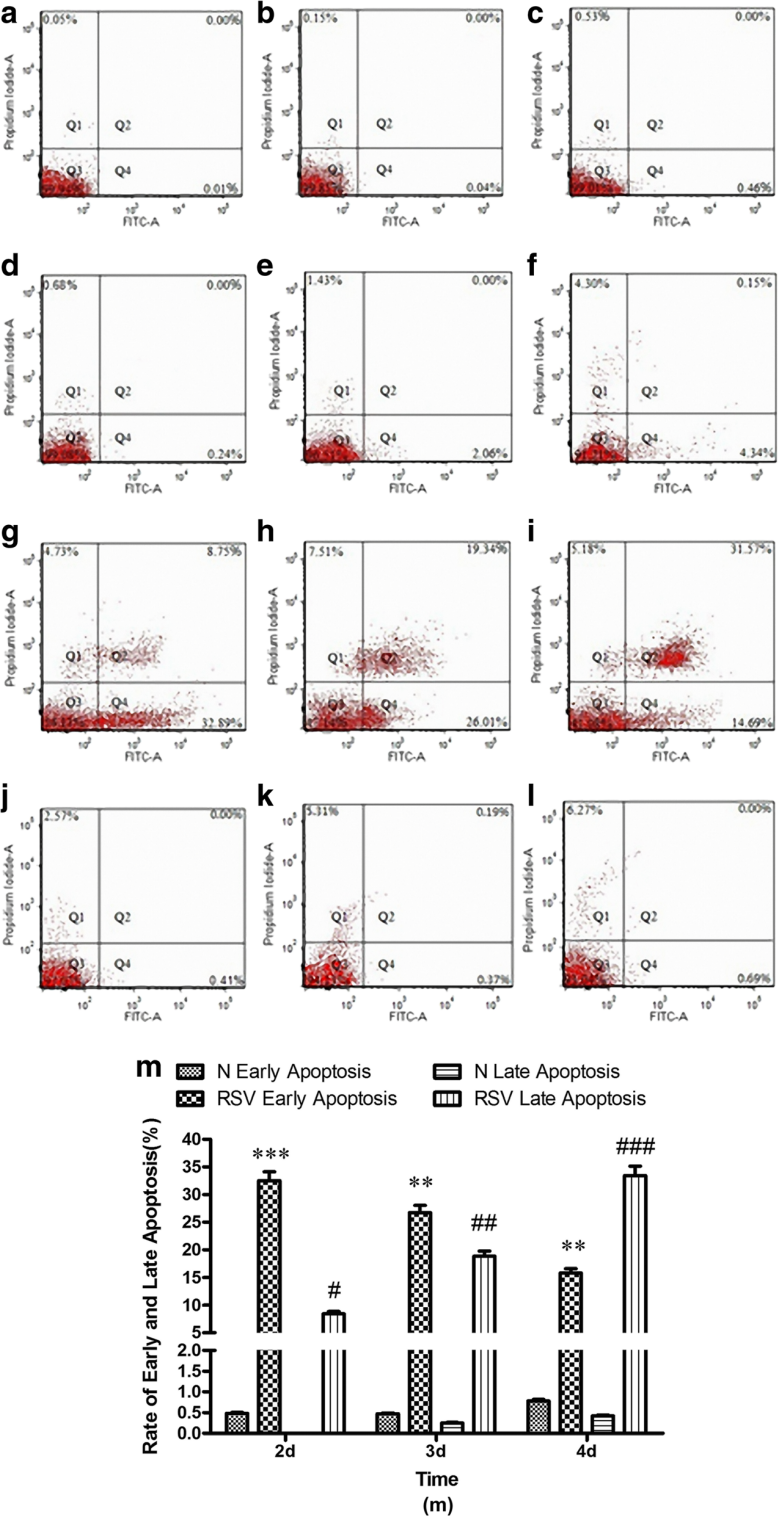
Apoptosis analysis of RSV-infected N2a neuronal cells. After RSV infection, N2a cells were stained with FITC, Annexin-V and PI and subjected to flow cytometry analysis. Panels **a**, **b**, **c**, **d**, **e**, **f**, **g**, **h**, and **i** represent the apoptotic statuses of N2a cells at 0 h, 1 h, 2 h, 4 h, 12 h, 24 h, 2 d, 3 d and 4 d after RSV infection, respectively. Panels **j**, **k**, and **l** represent the apoptotic statuses of uninfected N2a cells at 2 d, 3 d and 4 d, respectively. Data are expressed as the mean ± SEM, and all tests were run in triplicate (shown in fig. m). ^*^P < 0.05, ^**^P < 0.01, and ^***^P < 0.001 indicate the *P* values of early apoptosis compared with those of the uninfected control group. ^#^*P* < 0.05, ^##^*P* < 0.01, ^###^*P* < 0.001 indicate the P values of late apoptosis compared with those of the uninfected control group

To investigate changes in the apoptosis levels of both RSV-infected N2a cells and uninfected control cells, flow cytometry analysis was adopted using FITC-conjugated Annexin V and PI staining. As shown in Fig. 8, no early or late apoptotic cells were detected at 0 h pi, 1 h pi, 2 h pi, 4 h pi, 12 h pi or 24 h pi (Fig. 8a–f). At 2 d pi, the proportion of early apoptotic cells (Annexin V^+^, PI^−^) reached 32.89% in N2a cells, which was statistically significant, while the proportion of late apoptotic cells (Annexin V^+^, PI^+^) was 8.75%, statistically insignificant. At 3 d and 4 d pi, the early apoptosis ratios reached 26.01 and 14.69%, and the late apoptosis ratios reached 19.34 and 31.57%, respectively (Fig. 8g–i), both of which were statistically significant different compared with those in the uninfected control group. Additionally, necrosis and apoptosis were not apparent in the uninfected control group at 2 d pi, 3 d pi or 4 d pi (Fig. 8j–l) Therefore, from 2 d pi to 4 d pi, longer durations of RSV infection appeared to correlate with a gradual decrease in early apoptotic cells and a gradual increase in late apoptotic cells.

### Increased TLR3 and TLR7 expression in RSV-infected N2a cells

**Fig. 9:**
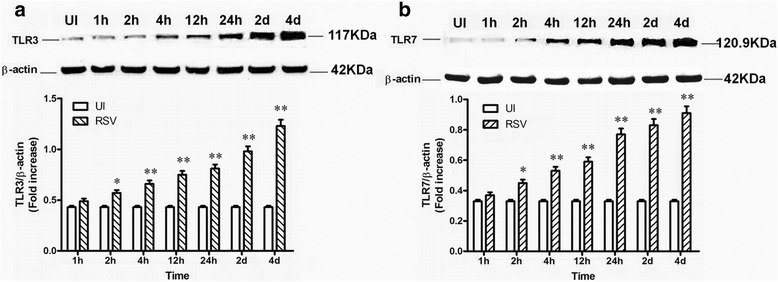
TLR3 and TLR7 protein expression in RSV-infected and uninfected N2a cells at different time points as determined by Western blot analysis. Lane 1: mock control; lanes 2–8: RSV-infected N2a cells at the time points 1 h pi, 2 h pi, 4 h pi, 12 h pi, 24 h pi, 2 d pi, and 4 d pi, respectively. The protein levels of TLR3 (**a**) and TLR7 (**b**) were enhanced as the duration of RSV infection was extended. In addition, the statistical significance of relative fold changes in TLR3 (**a**) and TLR7 (**b**) expression were evaluated using GraphPad Prism 5 software (GraphPad Software Inc.). Data are expressed as the mean ± SEM, and three independent experiments were run. ^*^P < 0.05, was considered statistically significant, and ^**^P < 0.01 was considered highly statistically significant compared with the uninfected control

To further evaluate the activating effects of RSV infection on intracellular TLRs, the protein expression levels of TLR3 and TLR7 in both the RSV-infected group and the uninfected control group were analyzed by Western blot. TLR3 protein expression in the RSV-infected group steadily increased from the starting time at 1 h pi (Fig. 9a) in a time-dependent manner compared with that in the uninfected control group (*P* < 0.01). Similarly, expression of the TLR7 protein was relatively low in the uninfected control group, and TLR7 protein expression in the RSV-infected group increased as the duration of RSV infection extended in a time-dependent manner. (Fig. 9b). This increase in the RSV-infected group was significantly different from that in the uninfected control group (*P* < 0.05).

### Increased IL-6 and TNF-α levels in the culture supernatants of RSV-infected N2a cells

**Fig. 10:**
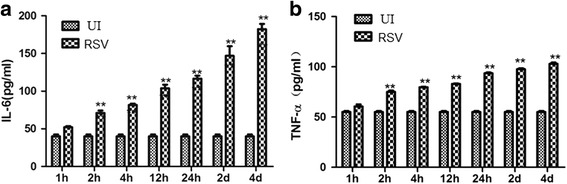
Expression levels of inflammatory cytokines in the culture supernatants of RSV-infected and uninfected N2a cells as measured by ELISA. The expression levels of IL-6 (**a**) and TNF-α (**b**) were enhanced as the duration of RSV infection was extended. UI indicates the uninfected control. Data are expressed as the mean ± SEM, and three independent experiments were run. ^*^P < 0.05 was considered statistically significant, and ^**^P < 0.01 was considered highly statistically significant compared with the uninfected control

RSV-induced encephalopathy is accompanied by neuronal cell damage and frequently altered expression levels of pro-inflammatory cytokines, such as IL-6, IL-8 and TNF-α. Therefore, variations in pro-inflammatory cytokine levels in the culture supernatants of virus-infected N2a cells potentially confirm the proliferation of viral infection in N2a neuronal cells. Thus, the protein expression levels of IL-6 and TNF-α in RSV-infected N2a neuronal cells were assessed by ELISA. IL-6 and TNF-α levels in the RSV-infected group were substantially elevated compared with that in the uninfected control group as the duration of RSV infection extended (*P* < 0.01), suggesting that encephalopathy-associated pro-inflammatory molecules are induced by RSV infection in N2a cells (Fig. 10).

## Discussion

RSV is the major pathogen responsible for acute lower respiratory infections in human infants [32], and Zlateva et al. suggested that RSV might cause neurological complications in infants [5]. In addition, some cases of RSV being potentially associated with clinical encephalopathy, coughing and runny nose symptoms in RSV-infected children who developed unconsciousness and hypotonia have been reported [33]. Furthermore, the RSV viral genome has been detected in the cerebrospinal fluid of children infected with RSV diseases [5, 34]. Because virus-induced encephalopathy may lead to sudden infant death syndrome [2, 3], determining whether RSV can infect neuronal cells or access children brains via the CNS is very important. Based on the report that coronaviruses (CoVs) may access the CNS by crossing the blood brain barrier (BBB**)** [35], herein, neuronal N2a cells were selected to test whether RSV can infect the CNS and induce neuronal damage.

Though many clinical cases of RSV-induced encephalopathy have been reported, relative experimental research was unfortunately seldom conducted, and the mechanisms underlying this disease have yet to be elucidated. Thus far, no effective treatments have been identified, and basic studies reporting virus-induced encephalopathy have been limited to the rabies and influenza viruses [10, 36]. Thus, in the current study, the pathogenesis of RSV-associated encephalopathy was preliminarily explored. Specifically, the roles of two membrane receptors, TLR4 and C23, in the process of RSV infecting neuronal cells were investigated.

First, the infectivity of RSV in N2a neuronal cells was assessed. As shown in Figs. 1 and 2, the number of NeuN-positive cells decreased, and their cellular and nuclear morphological characteristics were altered at 4 d after RSV infection, as determined by light and confocal microscopy. Additionally, expression of the RSV F protein in virus-infected N2a cells increased in a time-dependent manner (Fig. 3). These data confirmed that RSV can infect, propagate and proliferate in N2a cells.

Second, the co-localization and potential interactions of the RSV F protein with TLR4 and C23 were investigated by laser confocal microscopy and a co-immunoprecipitation assay. The F protein was found to co-localize with both the TLR4 and C23 receptors at 1 h pi (Figs. 5 and 6). The baseline level of C23 expression was slightly higher than that of TLR4, but both TLR4 and C23 protein expression were up-regulated from 1 h pi to 4 h pi, which is during the early stage of infection (Fig. 7). Interestingly, both TLR4 and C23 protein levels were down-regulated beginning 4 h pi (late stage of infection), suggesting that these two receptors may participate in the early recognition and modulation of viral entry into N2a cells. We also speculated that TLR4 and C23 have no additional functions after RSV is internalized, as the expression levels of these two membrane receptors were decreased after 4 h of infection. These data are consistent with those of the laser confocal microscopy and co-immunoprecipitation experiments. TLR4 expression in neurons of traumatic encephalopathy patients has also been reported to increase at early infection and decrease at late infection, and TLR4 is thought to play a vital role in inflammatory responses involving MyD88/NF-κB activation and initiate the TLR4/MyD88/NF-κB signaling pathway [26, 37], suggesting that TLR4 is very important for the ability of RSV to infect neuronal cells.

Third, because apoptosis may involve virus-cell interactions and induce cellular damage during the viral infection process, the apoptosis rates of N2a cells after RSV infection were measured in this study. Early apoptosis occurred at 2 d pi, and late apoptosis occurred as the duration of RSV infection extended. These findings demonstrated the presence of apoptotic lesions during the RSV infection process.

Fourth, because TLRs are expressed in both non-neuronal and neuronal cell types in the CNS and contribute to both infectious and non-infectious disorders [38], TLR3 and TLR7 are thought to be major mediators of virus-induced signaling pathways during RSV infection [39, 40]. TLR3 activation is associated with neuroinflammation and common CNS disease pathologies [41], and TLR7 is up-regulated upon chikungunya virus (CHIKV) infection and plays an important role in CHIKV pathogenesis in neuronal cells [42]. Moreover, TLR activation in dendritic and neuronal cells has been reported to drive a protective antiviral response [43, 44]. Thus, in this study, variations in TLR3 and TLR7 expression in RSV-infected N2a cells were determined. Both TLR3 and TLR7 protein levels continuously increased as the duration of RSV infection was extended, confirming the role of TLR-mediated innate immune responses in RSV pathogenesis.

The experiments of this study were performed in triplicate, and consistent results were achieved. However, our study does have some limitations. For example, regarding the receptor-mediated effects on virus invasion, only preliminary evidence was obtained. Regarding the roles of TLR4 and C23 in RSV-infected neurons, the RSV F protein was shown to co-localize with both TLR4 and C23, but their specific interactions and binding were not fully investigated. Further experiments, such as yeast two hybrid, GST pull-down and RNA interference experiments, are required to confirm the interactive activity of the RSV F protein with TLR4 and C23.

Based on the above experiments, RSV Long strain prolifically infects N2a neuronal cells and induces cell lesions, and TLR4 and C23 expressed on the cell membrane surface might mediate the viral entry process in N2a cells and further activate expression of the intracellular receptors TLR3 and TLR7. Simultaneously, TLR-associated downstream inflammatory factors, such as IL-6 and TNF-α, are released from N2a cells by RSV invasion. Notably, the results obtained herein from RSV-infected N2a cells are similar to those obtained from CHIKV virus–infected N2a cells [42].

## Conclusions

In summary, our data demonstrated that RSV prolifically entered and infected N2a neuronal cells, leading to the modulated expression of TLR4 and C23 as well as of TLR3, TLR7 and their downstream inflammatory factors, suggesting a direct induction of RSV-associated encephalopathy in infants by the RSV infection of neuronal cells. During these processes, the RSV F protein co-localized with TLR4 and C23 and thus may play an essential role in the RSV infection of N2a cells. Taken together, the results presented here may help elucidate the underlying pathology of RSV infection and suggest potential therapeutic targets for the control and prevention of RSV-induced human diseases.

## Abbreviations

C23: Nucleolin
CNS: Central nervous system
CoVs: Coronaviruses
CSF: Cerebrospinal fluid
FCS: Fetal calf serum
JEV: Japanese encephalitis virus
LPS: Lipopolysaccharides
MOI: Multiplicity of infection
N2a: Neuro-2a
PFUs: Plaque-forming units
pi: Post-infection
PI: Propidium iodide
PMSF: Phenylmethylsulfonyl fluoride
PVDF: Polyvinylidene fluoride
RSV: Respiratory syncytial virus
RT: Room temperature
SDS-PAGE: Sodium dodecyl sulfate-polyacrylamide gel electrophoresis
TLR4: Toll-like receptor 4
TLRs: Toll-like receptors
